# Solitary uterine metastasis of invasive lobular carcinoma after adjuvant endocrine therapy: a case report

**DOI:** 10.1186/s13256-014-0511-6

**Published:** 2015-02-14

**Authors:** Masafumi Toyoshima, Hideki Iwahashi, Takashi Shima, Atsushi Hayasaka, Takako Kudo, Hiromitsu Makino, Saori Igeta, Rui Matsuura, Nobuko Ishigaki, Kozo Akagi, Junko Sakurada, Hiroyoshi Suzuki, Kosuke Yoshinaga

**Affiliations:** 1 Department of Obstetrics and Gynecology, Sendai Medical Center, National Hospital Organization, 2-8-8, Miyagino, Miyagino-ku, Sendai, Miyagi 983-8520 Japan; 2 Department of Pathology, Sendai Medical Center, National Hospital Organization, Sendai, Japan; 3 Department of Obstetrics and Gynecology, Self-Defense Force Sendai Hospital, Sendai, Japan; 4 Department of Obstetrics and Gynecology, Sendai City Hospital, Sendai, Japan

**Keywords:** Anastrozole, Breast cancer, Immunohistochemistry, Invasive lobular carcinoma, Isolated uterine metastasis

## Abstract

**Introduction:**

Solitary uterine metastases from extragenital cancers are very rare. Breast cancer is the most frequent primary site of metastasis to the uterine corpus, with invasive lobular carcinoma more likely to spread to gynecologic organs than invasive ductal carcinoma.

**Case presentation:**

A 62-year-old postmenopausal Japanese woman was diagnosed with uterine leiomyomata more than 20 years ago and had been managed conservatively until menopause. Seven years prior to her presentation, she was diagnosed with breast cancer and underwent a partial resection of her right breast for stage IIA invasive lobular carcinoma. She underwent adjuvant chemotherapy, radiotherapy, and five years of anastrozole hormonal therapy. She presented with a growing uterine mass. Her tumor marker levels were markedly increased over the course of her follow-up, but a systemic examination revealed only a solitary uterine tumor. She underwent a total abdominal hysterectomy with bilateral salpingo-oophorectomy. A histopathological examination, including detailed immunohistochemistry, confirmed metastatic invasive lobular carcinoma, infiltrating both her uterine myometrium and fibroid tissue.

**Conclusion:**

We report a very rare metastatic pattern of invasive lobular carcinoma and demonstrate that gross cystic disease fluid protein-15 and mammaglobin are useful in the diagnosis of metastatic breast cancer.

## Introduction

The ovary and the vagina are the most frequent metastatic sites for both extragenital and genital primary neoplasms, but it is very rare for extragenital cancers to metastasize to the female reproductive tract [1]. Breast cancer is the most frequent extragenital origin of metastasis to the uterine corpus [2]; however, uterine involvement remains very rare. The most common sites of breast cancer metastasis are lung, bone, liver, and brain. Most uterine metastases are found at the time of autopsy [3] because uterine involvement by metastasis is a sign of end-stage disease. When compared with invasive ductal carcinoma (IDC) of the breast, the stage-matched prognosis is better for patients with invasive lobular carcinoma (ILC) [4]. However, ILC is more likely to spread to gynecologic organs [5,6].

Anastrozole, a third-generation aromatase inhibitor, has been shown to be superior to tamoxifen as adjuvant therapy for patients with breast cancer [7]. In addition, anastrozole is expected to be safer for the prevention of recurrence and metastasis, because tamoxifen increases the risk of endometrial cancer [8].

We report the case of a patient with enlarging uterine fibroids after menopause. Seven years prior, she had been treated for ILC with surgery, radiotherapy, and anastrozole hormonal therapy. She underwent a total hysterectomy, and a pathological examination at this time revealed a solitary metastatic ILC lesion involving the uterine myometrium and infiltrating a fibroid.

## Case presentation

A 62-year-old postmenopausal Japanese woman presented with a 23×12cm uterine mass. The mass was growing, and our patient reported abdominal compression. She had first been diagnosed with uterine leiomyomata more than 20 years previously and had taken gonadotropin-releasing hormone agonist therapy for six months. Her age at menopause was 51 years and at the age of 55 years, she was diagnosed with breast cancer and underwent a partial resection of her right breast. A pathological examination of the neoplasm revealed ILC, classified as pT2N1M0, stage IIA. Immunohistochemical staining was positive for estrogen receptors, progesterone receptors, and cerbB2, and 20% of the cells were positive for Ki-67. She received adjuvant radiotherapy, 50 Gy to her right breast and an additional 10 Gy for localized irradiation. Our patient also underwent adjuvant chemotherapy with six cycles of fluorouracil, epirubicin, and cyclophosphamide. In addition, she took anastrozole as hormonal therapy for five years after surgery.

**Figure 1:**
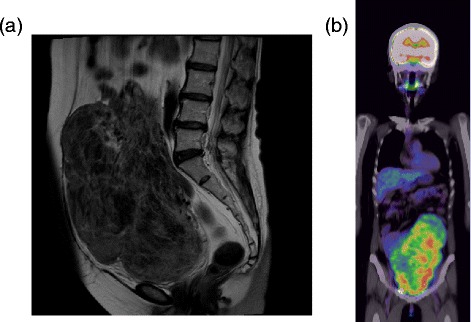
**Radiological images. (a)** Pelvic magnetic resonance imaging, T2-enhanced sagittal section, showing an irregularly enlarged uterine tumor. **(b)** Positron emission tomography-computed tomography showing strong uptake associated with the uterine tumor.

Twenty months after the therapy ended, she was noted to have elevated tumor markers (cancer antigen 15-3, 87.9IU/mL; carcinoembryonic antigen (CEA), 15.6ng/mL; National Cancer Center stomach 439, 120IU/mL). A diagnostic workup was undertaken that included computed tomography, magnetic resonance imaging (Figure 1a), and transvaginal ultrasonography. Her uterus was irregularly enlarged, measuring 23×12cm. Despite her postmenopausal status, the uterine leiomyomata had markedly enlarged since the previous examination two years prior. A systemic examination, including technetium 99m hydroxymethylene diphosphonate bone scintigraphy, positron emission tomography-computed tomography (Figure 1b), and upper endoscopy, revealed no other lesions. Endometrial sampling was technically impossible given the size of the mass.

**Figure 2:**
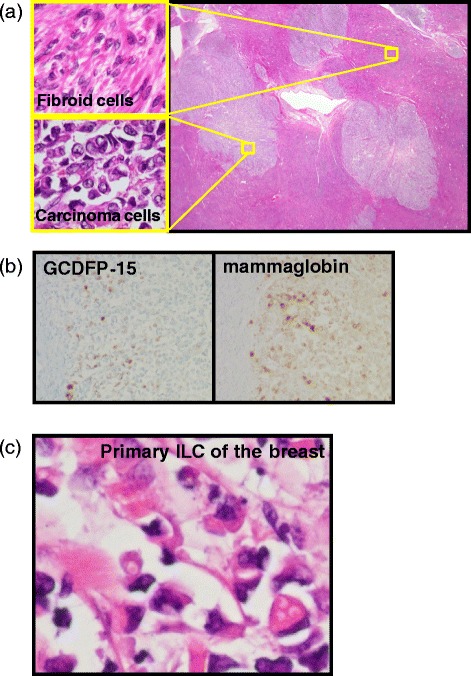
**Pathological microscopic examination. (a)** Clusters of malignant epithelial cells infiltrate the uterine fibroma. Staining: hematoxylin and eosin. Top left: magnified image of fibroid cells; bottom left: magnified image of carcinoma cells. **(b)** Immunohistochemical staining positive for gross cystic disease fluid protein-15 (GCDFP-15) and mammaglobin. **(c)** Hematoxylin and eosin staining of the primary breast cancer specimen demonstrates poor epithelial staining of the malignant cells with irregular nuclei. ILC, invasive lobular carcinoma.

Our patient underwent a total abdominal hysterectomy and bilateral salpingo-oophorectomy with the diagnosis of uterine malignancy, although it was unknown whether the tumor was primary or metastatic. A pathological examination revealed malignant cells with poor epithelial staining, along with irregular nuclei; these cells were infiltrating both her uterine fibroid tissue and her myometrium (Figure 2a). There was a small nest of malignant cells in the stroma of the atrophic endometrium. Immunohistochemical staining was positive for estrogen receptors, cytokeratin AE1/AE3, anti-cytokeratin CAM5.2, cluster of differentiation (CD) 10, CEA, cytokeratin (CK) 7, gross cystic disease fluid protein-15 (GCDFP-15), and mammaglobin (Figure 2b). The tumor was negative for progesterone receptors, CD45, myeloperoxidase, CD68, chromogranin A, synaptophysin, CD56, vimentin, desmin, smooth muscle actin, CK20, CDX-2, and E-cadherin. The previous breast tissue specimens were also examined, and malignant cells with a similar form to those seen in the uterine tumor were observed (Figure 2c). The histopathological diagnosis was metastasis of breast ILC to both the uterine leiomyoma and the myometrium. Our patient is currently undergoing exemestane hormonal therapy and remains without evidence of recurrent disease.

## Discussion

ILC accounts for about 10% of breast cancer in female patients [9]. The sites of metastatic spread in ILC differ from those of IDC: in the latter, the common metastatic sites are the lung, bones, liver, and brain; in ILC, the gastrointestinal tract, peritoneum, retroperitoneum, and gynecologic organs have been reported as metastatic sites [5]. Anastrozole is considered to be better for adjuvant therapy than tamoxifen, especially for postmenopausal patients with hormone-sensitive early breast cancer [7]. The anticancer effects of anastrozole have also been studied in endometrial cancers in a randomized pilot study [10]. Despite the fact that there is a wide range of metastatic sites possible in patients with ILC, it is extremely rare to see solitary uterine involvement after anastrozole therapy for the disease; just a few case reports have been published in the English literature [11-13]. These reports emphasize the usefulness of GCDFP-15, known for having high sensitivity and specificity for breast cancer, as a tumor marker at the metastatic site [11-13]. In our patient, the malignant epithelial cells within her uterus showed poor epithelial staining and had little cytoplasm and irregular nuclei. The pathological diagnosis was very difficult on the basis of hematoxylin and eosin staining alone: a wide differential diagnosis was possible, including a blood-derived tumor, a neuroendocrine tumor, a sarcoma, and small cell carcinoma. However, immunohistochemistry positivity for cytokeratin, estrogen receptors, progesterone receptors, GCDFP-15, and mammaglobin gave definitive information by which we could make the diagnosis of metastatic ILC to the uterus.

If metastatic infiltration involves the endometrial glands, then abnormal uterine bleeding is often the first symptom, and endometrial sampling allows the detection of malignant cells [14]. However, patients may often be asymptomatic when the infiltration affects only the myometrium, as in our patient. When tumors metastasize to the uterus, they involve only the myometrium in 64.5% of cases, both the myometrium and the endometrium in 32.7%, and only the endometrium in just 3.8% [1]. Our patient presented with abdominal distension but without abnormal bleeding. Furthermore, a huge uterine fibroid prohibited endometrial sampling. Hence, clinicians should keep in mind that metastasis of malignant cells to the uterus does not always result in ‘typical symptoms’ such as abnormal genital bleeding.

## Conclusions

We report a very rare metastatic pattern of ILC and demonstrate that detailed immunohistochemistry, including GCDFP-15 and mammaglobin, is useful in the diagnosis of metastatic breast cancer. It is important for clinicians to recognize that ILC and IDC have different patterns of metastasis and that an isolated uterine metastasis may occur after either anastrozole or tamoxifen therapy. A comprehensive gynecologic workup should be considered in patients with breast cancer who have uterine lesions.

## Consent

Written informed consent was obtained from the patient for publication of this case report and accompanying images. A copy of the written consent is available for review by the Editor-in-Chief of this journal.

## Abbreviations

CD: cluster of differentiation
CEA: carcinoembryonic antigen
CK: cytokeratin
GCDFP-15: gross cystic disease fluid protein-15
IDC: invasive ductal carcinoma
ILC: invasive lobular carcinoma
